# Risk factors of stunting (chronic undernutrition) of children aged 6 to 24 months in Mekelle City, Tigray Region, North Ethiopia: An unmatched case-control study

**DOI:** 10.1371/journal.pone.0217736

**Published:** 2019-06-10

**Authors:** Kidanemaryam Berhe, Omer Seid, Yemane Gebremariam, Almaz Berhe, Natnael Etsay

**Affiliations:** 1 Department of Public Health, Adigrat University, Adigrat City, Ethiopia; 2 Department of Nutrition, Mekelle University, Mekelle City, Ethiopia; 3 Department of Health Systems, Mekelle University, Mekelle City, Ethiopia; 4 Department of Midwifery, Mekelle University, Mekelle City, Ethiopia; 5 Department of Midwifery, Adigrat University, Adigrat City, Ethiopia; Public Library of Science, UNITED KINGDOM

## Abstract

**Introduction:**

In 2014, 159 million under 5 year-old children were stunted (suffered chronic undernutrition) worldwide. Identifying risk factors for stunting among 6 to 24 month-age children in Mekelle City is important for evidence-based interventions.

**Method:**

Case-Control study design was undertaken in 330 children, from January to February 2016. World Health Organization (WHO) anthropometric software and statistical package for social sciences version 20 were used for analysis. Logistic regression analysis was applied.

**Result:**

The following were identified as risk factors for stunting: mother’s lack of formal education (adjusted odds ratio (AOR = 6.4)), mother height less than 150cm (AOR = 4.2), mother with a body mass index less than 18.5 kg/m^2^ (AOR = 3.8), childbirth weight less than 2.5kg (AOR = 5.3), household with two and above under-five children (AOR = 2.9), a WHO diet diversity score < 4 (AOR = 3.2) and repeated diarrheal episodes (AOR = 5.3).

**Conclusion:**

The factors associated with stunting among children aged 6 to 24 months are no formal education in mother, mother height less than 150cm, low BMI of the mother, low birth weight, low WHO DDS, number of under 5 children in the household and repeated diarrheal episodes. Nutritional interventions should give emphasis to maternal education, maternal nutrition, childbirth weight, family size, diet diversity, and diarrheal diseases.

## Introduction

Stunting is a measure of chronic undernutrition and it is measured by length or height for age standard deviation score (z-score) [1]. In 2014,159 million under 5-year-old children were stunted worldwide. More than half of all stunted under-five children lived in Asia (57%) and more than one third lived in Africa (37%). Africa was the only region where the number of stunting among under five-year-old children increased over the past decade. By 2020 stunting prevalence in Africa is estimated to reach 40% with 70.2 million children affected [2]. The consequences of stunting in children are broad and include high morbidity and mortality, display of less exploratory behavior, higher anxiety, depression, poor health, adult short stature, chronic diseases later in life, poor intelligent quotient (IQ) level, poor cognitive function, and poor school achievements [3, 4].

The World Health Organization (WHO) has adopted a target of reducing the number of stunted children under the age of 5 by 40% by 2025 [5]. The most crucial time to meet a child’s nutritional requirement is in the first 1,000 days which is from conception up to the child’s second birthday. During this period (first 1000 days), the child has increased nutritional needs to support rapid growth and development, the child is more susceptible to infections, the child has high sensitivity to biological programming and the child is totally dependent on others for nutrition, care and social interactions [5, 6]. Similarly, the Federal Government of Ethiopia has been working to reduce stunting (to 26% by 2020) through different interventions [7] but in Ethiopia, the prevalence of stunting (chronic undernutrition) in under 5 children is a severe public health problem. According to the 2014 mini Ethiopia demographic and health survey (EDHS), nationally about 40% of under 5 children were stunted and the prevalence of stunting in Tigray Region was 46%, which was above the national stunting prevalence. This high prevalence and poor progression towards solving stunting might be due to a lack of evidence on the risk factors that affect stunting and the lack of evidence-based interventions [8, 9]. Family income, maternal educational level, family size, mother’s height <145cm and gestational age of the child were found as risk factors for stunting in children according to studies undertaken in northeastern Brazil, Myanmar, and Indonesia [10,11,12]. Findings of research conducted in Jhangara town and Egypt identified that no exclusively breastfeeding for at least four months of age, age at weaning, discarding the colostrums, giving complementary feeding too early, complementary feeding less than four times per day and diet diversity score below WHO standard was significantly associated with stunting but there was no association between stunting and bottle feeding [13, 14].

In Tigray region as well as the study area (Mekelle City), the risk factors for stunting among 6–24 month -age children have not been studied. Identifying the risk factors for stunting among 6 to 24 month-age children is essential to overcome the problem of stunting and its consequences. Again it is an important step in the study area to inform the design of different interventions, strategies, policies and resource allocation at different levels by public health bodies.

## Methods and materials

The study was conducted after approval from the college of health sciences research review committee, Mekelle University. An official letter was taken from the school of public health to Tigray Regional Health Bureau and health facilities. Permission from Tigray Regional Health Bureau, Mekelle sub city health offices and health facilities were obtained. Informed consent was taken and all data were handled confidentially. The right of the participants to withdraw at any time was respected. There was no procedure that could put the participant on risk and these all were explained before interviewing using the local language (Tigrigna). Mothers or caregivers were counseled on child and maternal nutrition, sanitation, and hygiene practices.

### Study area, study period and study population

This study was conducted from January 2016 to February 2016 in Mekelle City, Tigray Region, Ethiopia. Mekelle City is the capital city of Tigray Region and is located 780 kilometers to the North of Addis Ababa (the capital city of Ethiopia). The total population of the city is 340,859 with 49% males and 51% females while the total number of children with age of 6 months to 2 years was 20,452 in the year 2016 which was projected from the Ethiopia Central Statistical Agency 2007. All children 6 to 24 month-age who visited health facilities in Mekelle City were the source population.

#### Study design

Health facility-based unmatched case-control study design was applied.

### Eligibility criteria

Children 6 to 24 month-age with their mothers or caregivers who visited health facilities during the study period and who lived in Mekelle City for at least 6 months were included in the study. Children with a visible deformity, children without their mother or caregiver, children for whom the mother or caregiver didn’t know the exact age of the child were excluded from this study.

### Sample size determination

The sample size was calculated using EPI INFO version 3.5.1. The exposure considered was exclusive breastfeeding for more than six months. The proportion of this exposure in cases group and in controls group was 11.6% and 2.5% respectively [15]. Assuming of 95% CI, 80% power, control to case ratio of 2:1 and 10% for non-response rate, the total sample size was 330 (220 controls and 110 cases).

### Sampling techniques

A simple random method was used to select 6 health centers from 9 health centers. Consecutive sampling technique was used to select children of 6 to 24 month-age with their mothers or caregivers during data collection until the required sample size was attained. The total sample size from each selected health center was calculated based on the population proportion size (PPS) of the catchment areas of the health centers. During data collection, for each case, two controls were selected and this procedure was continued until the calculated sample size was attained.

### Data collection tools and procedures

Data were collected by face to face interview using a structured questionnaire adapted from standard questionnaires of WHO and UNICEF, Ethiopia demographic and health survey and previous similar literature [4,8,9,16]. Variables like income and the list of local foods were adjusted. For example in this study income was categorized as <512.6 birr, between 512.6 and 1498.5 birr and >1498.5 birr. Nursing professionals who had training or experience on anthropometric measurement and interviewing were recruited as data collectors. Similarly, degree nurses who had more experience or training on anthropometric measurement and nutritional assessment were supervisors. The questionnaire was designed to enable to acquire information concerning socio-demographic and economic factors, health care factors, child feeding practices factors, sanitation, and water factors. Twenty four hours dietary recall method was used to assess child diet diversifying score and meal frequency by using a checklist adapted from WHO (2010) guideline. The diet diversity score (DDS) indicates the adequacy and quality of the diet for child growth, development, and health. The length of children, height, and weight of mothers were measured using anthropometric methods. The length of the child was measured in a recumbent position to the nearest 0.1 cm using a standard lying board with a detachable sliding foot piece. Mother height was measured in stand position to the nearest 1 cm. Mothers’ weight was measured to the nearest 0.1kg.

### Data quality assurance

The questionnaire was prepared in English and then translated to the local language (Tigrigna). It was translated back to English to ensure consistency. Before the actual data collection, the questionnaire was pre-tested in 5% of the sampled population in Wukro town (out of the study area). Data collectors and supervisors were trained for two days to have a common understanding of the questionnaire, objective of the study, how to interview and how to perform the anthropometric measurements. Weighing scales were calibrated with known weight object regularly. The scale indicator was checked against zero reading after weighing every mother. There was strict supervision on the data collection process, consistency and completeness of questionnaires on a daily basis. The overall data collection process was controlled by the principal investigator. Filled questionnaires were checked and cleaned.

### Data analysis

Data were checked for completeness, edited, coded and entered to SPSS version 20 for analysis. Outcome variable was dichotomized into cases = 1 and controls = 0. After cleaning data for inconsistencies and missing values, descriptive statistics were done. Bivariate logistic regression was performed and variables with a p-value < 0.25 were transferred to multivariate logistic regression to identify the risk factors. In the multivariate logistic regression analysis, variables with p-value ≤0.05 were taken as statistically significant factors. Adjusted odds ratio with its 95% confidence intervals was considered to assess the association of the factors and stunting. Model goodness of fitness was assessed using Hosmer and Lemeshow test and the p-value for the Hosmer and Lemeshow test was 0.43 which suggests a good model (if the p-value is >0.05, the model fits; If p-value ≤0.05, the model is unfit). Multicollinearity between independent variables was checked and there was no collinearity.

## Result

### Socio-demographic and economic characteristics of study participants

A total of 330 children 6 to 24 month-age with their mothers or caregivers were included in this study (110 cases and 220 controls), the response rate was 100%. In the cases group, 48(43.6%) mothers were in the age of 30–39 years while in the controls group, 106 (48.2%) mothers were in the age of 30–39 years old. In both cases group and controls group, the majority of the mothers or caregivers (88.2% of cases and 93.2% of controls) were Tigrean in ethnicity. A majority of the cases group (84.5%) and controls group (88.2%) were orthodox Christian in religion. Above one third (34.5%) of mothers or caregivers in cases group and 6.4%, mothers or caregivers in the controls group had no formal education. In addition to this, 20 (18.2%) fathers in the cases group and 23(10.5%) fathers in the controls group had no formal education. A majority of cases’ (78.5%) and controls’ (90.7%) mothers’ height was 150cm and above. One-fifth (20.6%) of mothers in cases group and 15 (6.9%) mothers in the controls group were underweight (BMI<18kg/m^2^) (Table 1).

**Table 1:** Socio-demographic characteristics of participants in Mekelle City, Tigray Region, Ethiopia, 2016.

Variables	Case = 110 Number (%)	Control = 220 Number (%)
Child age in months		
6–8	13(11.8)	29(13.2)
9–11	20(18.2)	59(26.8)
12–17	48(43.6)	75(34.1)
18–24	29(26.4)	57(25.9)
Sex of child		
Female	51(46.4)	115(52.3)
Male	59(53.6)	105(47.7)
Marital status of mother/caregivers		
Married	98(89.1)	201(91.4)
Window/divorced	12(10.9)	19(8.6)

### Water and sanitation related characteristics of participants

About 88(80.7%) of children from cases group and174 (79.1%) children from controls group were found in households with a flush to sewage or septic tank type of toilet. A majority of the households for cases group (88.2%) and controls group (92.7%) had dwelling piped water supply as the main source of drinking water. Only 67(60.9%) mothers or caregivers from cases group and 148(67.3%) mothers or caregivers from controls group wash their hand at all critical times (after toilet, before food preparation or child feed, after child cleaning or after work). About 86(78.2%) cases and 180(81.8%) controls had a separated kitchen in their house (Table 2).

**Table 2:** Water and sanitation factors of participants in Mekelle City, Tigray Region, Ethiopia, 2016.

Variables	Case = 110, Number (%)	Control = 220, Number (%)
**Hand washing using**		
Only water	6(5.5)	14(6.4)
Sometimes with soap	27(24.5)	50(22.7)
Always with soap	77(70)	156(70.9)
**Method for Waste disposal**		
By municipal	97(88.2)	204(92.7)
Buried	6(5.5)	6(2.7)
Dumped in street/open space	7(6.4)	8(3.6)

### Health care related characteristics

About 50(45.5%) mothers in cases group and 102(46.4%) mothers in the controls group had 4 and above antenatal care (ANC) visits. Almost all mothers in both cases group (96.4%) and controls group (97.7%) gave birth (for this baby) in health institutions. Around one fourth (26.4%) of children from cases group and 5.9% of children from controls group had less than 2.5kg birth weight. About a quarter (25.5%) of cases and 29.5% of controls had postnatal care (PNC) follow up. 104(94.5%) of the cases and 213(96.8%) of the controls had completed and/or on right truck for immunization. Concerning child illness, about 35(31.8%) children from cases group and 21(9.5%) children from controls group had repeated diarrheal diseases.

### Child feeding practices related characteristics

About two-thirds (62.7%) mothers or caregivers in cases group and 69.5% mothers or caregivers in the controls group had received information concerning child feeding practices from health workers. In cases group, 48(43.6%) mothers and in the controls group,111(50.5%) mothers made decisions on money for livings. A majority of children in cases group (93.5%) and controls group (96.8%) had fed colostrums. About 66(61.7%) of children from cases group and 180(83.7%) of children from controls group were in exclusive breastfeeding for 6 months. 29(26.6%) of children in cases group and 23(10.6%) of children in the controls group started complementary feeding at the age of less than 6 months. Diet diversification score (DDS) in cases group was less than 4 in 59(57.8%) of the children while 137(71%) children in the controls group had 4 and above DDS.

### Risk factors for stunting among children of 6 to 24 month-age

In multivariate logistic regression analysis, only seven factors remained significantly associated with stunting (p-value ≤ 0.05). To avoid the risk of overfitting of the multivariate model, researchers select the most significant variables in bivariate logistic regression analysis and based on the available literature and theoretical knowledge. Interaction test was done and there was no interaction among the significant factors for stunting. The proportion of mothers with no formal education was significantly higher in cases group (stunted) compared to controls group (adjusted odds ratio (AOR) = 6.4; 95% confidence interval (CI): 2.02, 24.6). The proportion of mothers with a height less than 150 cm was higher in cases group than the controls group (AOR = 4.2; 95%CI: 1.9, 11.9). Mothers with a BMI of less than 18.5 kg/m^2^ were higher in cases group compared to the controls group (AOR = 3.8; 95%CI: 1.92, 20.1). Birth weight of less than 2.5kg was found to be a risk factor for stunting (AOR = 5.3; 95%CI: 2.1, 19.8). Households which had two and above under-five children were higher in cases group as compared to the controls group (AOR = 2.9; 95%CI: 1.4, 6.4). A statistically significant association was found between a WHO diet diversity score (DDS) of less than 4 and stunting (AOR = 3.2; 95%CI: 1.9, 16.4). The proportion of children who had repeated diarrheal episodes was higher in cases group as compared to the controls group (AOR = 5.3; 95%CI: 2.3, 19.1) (Table 3).

**Table 3:** Factors independently associated with stunting among 6 to 24 month-age children in Mekelle City, Tigray Region, Ethiopia 2016. (Result of Bivariate and Multivariate logistic regression analysis).

Variables	Case Number (%)	Control Number (%)	Crud OR(95%CI)	Adjusted OR(95% CI)
**Mother education**				
No formal education	38(34.5)	14(6.4)	9.4(4.47,19.6)**	6.4(2.02,24.6)**
1^0^ school	19(17.3)	53(24.1)	1.24(0.63,2.4)	3.1(0.9,7.2)
2^0^ school	24(21.8)	53(24.1)	1.56(0.83,2.95)	4.7(2.3,15.6)
Above2^0^ school(ref.)	29(26.4)	100(45.5)	1	1
**Mother height**				
<150 cm	23(21.5)	20(9.3)	2.68(1.4,5.1)**	4.2(1.9,11.9)**
≥150 cm(ref.)	84(78.5)	196(90.7)	1	1
Birth weight in kg**				
<2.5	29(26.4)	15(5.5)	5(2.54,9.94)**	5.3(2.1,19.8)**
2.5-4(ref.)	70(63.6)	182(82.7)	1	1
>4	6(5.5)	13(5.9)	1.2(0.44,3.3)	4.1(1.8,27.5)
**Number of children under 5 years of age in the household**				
1(ref.)	49(44.5)	155(70.5)	1	1
≥2	61(55.5)	65(29.5)	2.97(1.85,4.77)**	2.9(1.4,6.4)*
**DDS**				
<4	59(57.8)	56(29)	3.4(2,5.5)**	3.2(1.9,16.4)**
≥4(ref.)	43(42.2)	137(71)	1	1
**Mother BMI in kg/m**^**2**^				
<18.5	21(19.6)	15(6.9)	3.4(1.7,6.9)**	3.8(1.92,20.1)*
18.5–24.9(ref.)	81(75.7)	187(86.6)	1	1
≥25	5(4.7)	14(6.5)	0.8(0.3,2.4)	1.3(0.95,9.7)
**Repeated previous illness**				
Not ill(ref.)	46(41.8)	170(77.3)	1	1
Respiratory infection	21(19.1)	23(10.5)	3.5(1.75,6.79)**	3.8(1.82,21.6)*
Diarrhea	35(31.8)	21(9.5)	6.3(3.3,11.86)**	5.3(2.3,19.1)**
Fever	8(7.3)	6(2.7)	5.67(1.92,16.75)**	4.9(1.01,34.2)
Father Education				
No formal education	20(18.2)	23(10.5)	3.17(1.54,6.5)**	3.8(1.54,17.7)
1^0^ school	29(26.4)	43(19.5)	2.35(1.28,4.32)**	2.6(1.02,11.4)
2^0^ school	28(25.5)	40(18.2)	2.44(1.31,4.53)**	2.9(1.03,8/5)
Above 2^0^ school(ref.)	33(30)	114(51.8)	1	1
Duration of exclusive breastfeeding				
<4 months	12(11.2)	7(3.2)	4.4(1.6,12.2)**	4.5(0.92,24.5)
4–5 months	24(22.4)	21(9.6)	3.8(1.9,7.6)**	1.94(0.74,8.7)
6 months(ref.)	66(61.7)	180(82.6)	1	1
>6 months	5(5.3)	9(4.1)	1.7(0.56,5.4)	2.9(0.84,18.4)
Age at complementary feeding starting				
<6 months	29(26.6)	23(10.6)	2.98(1.6,5.5)**	1.83(0.65,8.6)
6–8 months(ref.)	80(73.4)	189(86.7)	1	1
Income				
<512.6 birr	28(25.5)	15(6.8)	6(2.98,12.1)**	1.85(0.7,8.6)
512.6–1498.5 birr	31(28.2)	41(18.6)	2.4(1.39,4.27)**	1.7(0.8,8.4)
>1498.5 birr(ref.)	51(46.4)	164(74.5)	1	1

* = P-value≤0.05

** = p-value<0.01, (ref.) = reference, OR = Odd Ratio, CI = Confidence Interval

## Discussion

In this study maternal formal education was found to be associated with child stunting. The proportion of mothers with no formal education was higher in cases group (stunted children) compared to the controls group (AOR = 6.4; 95% confidence interval (CI): 2.02, 24.6). A similar finding was observed in studies conducted in Ethiopia, Northeastern Brazil, Myanmar, Indonesia, Bangladesh (Dhaka City), Palestine, Libya, Medebay Zana woreda, shire indasilassie (Tigray Region), Urmia (Northwest of Iran) [8,10,11,12, 16,17,18,19, 20,21,22] in which mother educational status was found to be associated with child stunting. Giving attention to children, good care practices, utilization of accessible health care services are influenced by maternal educational level which in turn affects stunting and other health-related issues. Maternal education influences the preparation, procurement, and selection of nutritious foods for themselves and their children. Moreover, maternal education increases women’s knowledge and attitude to act on new information related to nutrition and health [23].

The proportion of mothers with height less than 150cm was higher in cases group as compared to the controls group (AOR = 4.2; 95%CI: 1.9, 11.9). This finding is consistent with studies done in Northeastern Brazil, Myanmar, Indonesia, Ethiopia and Sub-Saharan Africa [10, 11, 12, 16, 24]. Stunted growth can be passed on to the next generation by the intergenerational cycle of malnutrition. The short stature of the mother and maternal undernutrition increases the risk of intrauterine growth retardation (IUGR) [24, 25]. Again maternal short stature can restrict uterine blood flow, the growth of the uterus, placenta, and fetus which leads to intrauterine growth retardation and child stunting. This study showed that mothers with a BMI of less than 18.5kg/m^2^ were higher in cases as compared to controls (AOR = 3.8;95%CI: 1.92, 20.1). This finding is in line with studies done in Ethiopia, shire Indasilassie [16, 21] which identified mother low BMI (<18.5kg/m^2^) was a risk factor for child stunting. Poor maternal nutrition during pregnancy and breastfeeding can lead to stunted growth of their children. Women who are underweight during pregnancy and even before pregnancy will have reduced amino acid transport through the placenta to the fetus which contributes to fetal growth reduction and to have stunted children which perpetuate the intergenerational transmission of stunting [26].

Birth weight of less than 2.5kg was found to be a risk factor for child stunting (AOR = 5.3; 95%CI: 2.1, 19.8). Similar observations were seen in studies done in Ethiopia, shire Indasilassie, Urmia (Northwest of Iran) [16, 21, 22]. Weight at birth is a strong predictor for size in later life because most low birth weighted infants do not catch-up to normal size during childhood. Having low weight at birth has a profound adverse effect on the health and development of the neonate. In undernutrition child, there is a reduction in key hormones responsible for growth, such as insulin-like growth factor-1(IGF-1) and thyroid hormones which leads to lower linear growth. In addition, there is a reduction in anabolic events in insulin-dependent tissue synthesis, resulting in lower lean body mass and impaired bone growth [27,28].

Households which had two and above under 5 children were higher in cases group than controls group (AOR = 2.9; 95%CI: 1.4, 6.4). This finding is similar with the study done in Meskan district (Gurage zone, Ethiopia) which found that participants living in households with a high number of under-five children were more likely to develop stunting than those living in households with least (one) number of under-five children [15]. Mothers who had many under-five children (≥2) will have less time to care for each child than mothers who had one child. In addition to this, there could be more competition and sharing of available foods. It was found that diet diversity score (DDS) of less than 4 was statistically significant with child stunting (AOR = 3.2; 95%CI: 1.9, 16.4). This finding is in line with study findings in Ethiopia [8, 16]. Inadequate complementary feeding and a general lack of vital nutrients besides pure caloric intake is one cause for stunted growth. Children need to be fed diets which meet the minimum requirements (four) in terms of diet diversity in order to prevent undernutrition [26,28].

The proportion of children who had repeated diarrhea episodes in the past two weeks were higher in cases group as compared to the controls group (AOR = 5.3; 95%CI: 2.3, 19.1). This finding is consistent with findings of study done in Northeastern Brazil, Jhangara town, Egypt, Gurage zone, Dhaka City, Johannesburg, Vietnam, Mekakel woreda (East Gojam), Bule Hora district (Ethiopia) [10, 13, 14, 15, 17, 29, 30, 31, 32] by which they identified infection especially diarrhea and pneumonia as risk factors for child stunting. Other than receiving poor nutrient diet, this period (6–24 months) is associated with increased exposure to infections associated with taking other fluids (non-breast milk) and/or solids as well as ingestion of contaminated materials as the children start exploring their environment. The infection affects children's nutritional status by diminishing appetite, reducing nutrient absorption, increase metabolic requirements and increasing nutrient losses [30]. There is a reciprocal relationship with diarrhea leading to undernutrition and undernutrition predisposing to diarrhea. Undernourished children have more severe diarrhea episodes and a child with diarrhea can become undernourished. Child gender and bottle feeding were not associated factors in this study which is in line with study findings in Indonesia, Jhangara town, Egypt and Palestine [12, 13, 14, 18]. But bottle feeding was a risk factor in a study conducted in Meskan district (Gurage Zone) and West Gojam [15, 33] this difference might be due to the variation in study area and study period. The limitations of this study include possibility of recall bias and the use of DDS (Diet Diversifying Score) which might not reflect the children exact eating habits. In addition, an unmatched case-control study design was implemented where each case was not paired individually with a control according to background variables, while we tried to control confounders as part of the analysis, potential confounders may remain. The study design does not allow a determination of the time period between the factors studied and the onset of stunting.

## Conclusion

The factors associated with stunting among children aged 6 to 24 months are no formal education in mother, mother height less than 150cm, low BMI of the mother, low birth weight, low WHO DDS, number of under 5 children in the household and repeated diarrheal episodes. Nutritional interventions should give emphasis to maternal education, maternal nutrition, childbirth weight, family size, diet diversity, and diarrheal diseases.

## Abbreviations

ANC: Ante Natal Care
BMI: Body Mass Index
EDHS: Ethiopia Demographic Health Survey
LAZ: Length for Age Z score
PNC: Post Natal Care
SD: Standard Deviation
SPSS: Statistical Package for Social Science
UNICEF: United Nations International Children Emergency Fund
USAID: United States Agency for International Development
